# Long-Term Mild Heat Causes Post-Mitotic Pollen Abortion Through a Local Effect on Flowers

**DOI:** 10.3389/fpls.2022.925754

**Published:** 2022-07-11

**Authors:** Jiemeng Xu, Stuart Y. Jansma, Mieke Wolters-Arts, Peter F. M. de Groot, Martijn J. Jansen, Ivo Rieu

**Affiliations:** Department of Plant Systems Physiology, Radboud Institute for Biological and Environmental Sciences, Radboud University, Nijmegen, Netherlands

**Keywords:** long-term mild heat, oxidative stress, pollen development, tapetum development, tomato (Solanum lycopersicum)

## Abstract

Crop reproductive success is significantly challenged by heatwaves, which are increasing in frequency and severity globally. Heat-induced male sterility is mainly due to aborted pollen development, but it is not clear whether this is through direct or systemic effects. Here, long-term mild heat (LTMH) treatment, mimicking a heatwave, was applied locally to tomato flowers or whole plants and followed up by cytological, transcriptomic, and biochemical analyses. By analyzing pollen viability, LTMH was shown to act directly on the flowers and not *via* effects on other plant tissue. The meiosis to early microspore stage of pollen development was the most sensitive to LTMH and 3 days of exposure around this period was sufficient to significantly reduce pollen viability at the flower anthesis stage. Extensive cytological analysis showed that abnormalities in pollen development could first be observed after pollen mitosis I, while no deviations in tapetum development were observed. Transcriptomic and biochemical analyses suggested that pollen development suffered from tapetal ER stress and that there was a limited role for oxidative stress. Our results provide the first evidence that heat acts directly on flowers to induce pollen sterility, and that the molecular-physiological responses of developing anthers to the LTMH are different from those to severe heat shock.

## Introduction

The global surface temperature has risen significantly compared to the pre-industrial era and will continue to increase. As a result of this, heatwaves are occurring more frequently and becoming more severe, in terms of duration and temperature increment (IPCC, 2018). Temperatures higher than the ranges that plants are adapted to may result in heat stress, causing a decline in fitness and crop yield (Zhao et al., 2017). To maintain food security, there is an urgent demand to develop crop genotypes resilient to the warming climate (Ortiz-Bobea et al., 2021). While many biological processes in the plant are adversely affected by high temperatures, sexual reproduction and, in particular, pollen development are regarded as the most vulnerable in many species (Satake and Yoshida, 1978; Saini and Aspinall, 1982; Hedhly et al., 2009; Rieu et al., 2017; Begcy et al., 2019). This also applies to tomato, one of the main vegetable crops globally (Iwahori, 1965; Müller and Rieu, 2016).

Pollen development is a well-conserved process among flowering plants. In brief, during microsporogenesis, pollen mother cells (PMCs) differentiate and undergo meiosis to form tetrads of spores. Next, during microgametogenesis, microspores are released from the tetrads and undergo vacuolisation, nuclear polarization, and mitosis to form bicellular gametophytes. The gametophytes then mature and thereafter give rise to pollen grains ready for fertilization (McCormick, 2004). The developing pollen, in particular during the stages from meiosis to mitosis, receives nutrients, enzymes, and wall components from the surrounding sporophytic tissue layer, the tapetum (Lei and Liu, 2020).

A sudden heat shock and very high temperatures are well known to affect various processes during pollen development, depending on the timing of exposure. During meiosis, these types of heat stress affect recombination and cytokinesis (Ning et al., 2021). When experienced slightly later, it may result in abortion of tetrads and developing microspores, impair pollen wall deposition, and disturb tapetum development and function (Iwahori, 1965; Djanaguiraman et al., 2014; Song et al., 2015). Bicellular pollen can also be affected but are less sensitive (Frank et al., 2009; Jegadeesan et al., 2018). Anthers and pollen cells initiate a cytoplasmic heat shock response upon experiencing such high temperatures, suggesting the occurrence of stress from unfolding proteins (Frank et al., 2009; Giorno et al., 2010; Chaturvedi et al., 2015; Yang et al., 2015; Fragkostefanakis et al., 2016a; Mu et al., 2017). Indeed, an attenuated heat stress response (HSR) due to knockdown of *HSFA2* reduced tomato pollen tolerance to heat shock, while higher tolerance of male fertility to heat shock has been correlated to higher heat shock protein accumulation in some genotypes (Frank et al., 2009; Fragkostefanakis et al., 2016b; González-Schain et al., 2016; Pham et al., 2020). At the same time, the anthers and pollen suffer from oxidative stress, as evident from increases in reactive oxygen species (ROS) and ROS damage, as well as induction of antioxidants and the ROS scavenging machinery (Frank et al., 2009; Paupière et al., 2017; Djanaguiraman et al., 2018; Zhao et al., 2018).

Pollen development is also sensitive to heatwave-like temperature profiles, characterized by more mildly elevated temperatures, sustained for multiple days (long-term mild heat; LTMH). Several studies have found that LTMH exposure is most detrimental if it includes the time of meiosis/early microspore development (e.g., Ahmed et al., 1992; Sato et al., 2000; Porch and Jahn, 2001; Erickson and Markhart, 2002), suggesting some similarity to the effect of severe heat shock. However, it is not clear to what extent the cellular and physiological damage to anthers caused by the two types of heat is comparable, for example, whether cytoplasmic protein folding stress and ROS accumulation play a similar important role. Recent mild heat experiments in Arabidopsis suggest that the unfolded protein response (UPR) in the endoplasmic reticulum may be important in situations of milder heat (Deng et al., 2016; Feldeverd et al., 2020; Yamamoto et al., 2020). Transcriptomic analyses indicate a response to ROS upon LTMH exposure, but to what extent ROS contributes to cellular damage is not known (Bita et al., 2011). Regarding anther and pollen physiology, several studies indicate that LTMH exposure affects carbohydrate metabolism at the maturing pollen stage, although the reported effects on starch, sucrose, and hexose concentrations are not always the same, within and between species (Aloni et al., 2001; Pressman et al., 2002; Firon et al., 2006; Sato et al., 2006; Jain et al., 2007; Wang et al., 2020).

To be able to better understand the effects of LTMH on pollen development, detailed insight into the plant tissues and developmental stages that are affected is needed. Here, we used tomato, an important vegetable crop that increasingly often suffers from high-temperature stress, to investigate which plant tissue is affected by LTMH and which stage of pollen development is most sensitive to LTMH. We then determined the minimum period of LTMH exposure that reduces pollen viability at anthesis and compared the developmental progression of pollen and tapetum after control and a 4-day LTMH through thorough cytological and transcriptomic analysis. Finally, we evaluated the contribution of oxidative damage to pollen failure by analyzing lipid peroxidation levels, as well as ROS scavenging gene expression and enzyme activities.

## Materials and Methods

### Plant Cultivation and Treatment

Solanum lycopersicum cultivar Micro-Tom (obtained from the National BioResource Project, Japan; accession TOMJPF00001) was used in this study, except for the temperature-controlled airbag experiment, which was performed with cultivar Money Maker. Seeds were sown on commercial soil (Lentse Potgrond number 4, Horticoop B.V., Katwijk, The Netherlands) and covered with a thin layer of vermiculite. Two weeks later, seedlings were transplanted into the same soil supplemented with 4 g L^−1^ fertilizer (Osmocote exact standard 3–4 M, Everris International B.V., Geldermalsen, The Netherlands). During germination and the seedling growth period, Micro-Tom plants were kept in cabinets under controlled temperature conditions (CT; 25 °C/19 °C day/night; 12 h/12 h photoperiod, ~200 μmol s^−1^ m^−2^ provided by Philips Green Power LED DR/B/FR 120 lamps; 60% relative humidity). Upon flowering, approximately 1 month after sowing, plants were either maintained at CT or subjected to long-term mild heat (LTMH; 33 °C/27 °C, day/night). Similarly, Money Maker plants were grown in growth chambers, under control conditions (25 °C/19 °C day/night; 14 h/10 h photoperiod, ~300 μmol s^−1^ m^−2^ provided by Philips D-Papillon daylight spectrum 340W lamps; 70/80% relative humidity) or LTMH (31 °C/25 °C). For the airbag experiment, single trusses were contained in near-airtight 1-L zip-lock bags, which were flushed with the help of an air pump (1 L min^−1^). The air was derived directly from the room or first passed through a water bath to obtain a stable temperature of T+5 °C (“heated”) or T-6 °C (“cooled”) inside the bag.

### Pollen Viability Assay

The viability of mature pollen collected from freshly open flowers was determined by an *in-vitro* germination assay. In brief, anthers from the flowers were cut into four slices and rehydrated in 1.5-ml Eppendorf tubes for 30 min. Afterwards, pollen were incubated for 1.5 h in 0.5-ml germination medium (25% [w/v] PEG 4,000, 5% [w/v] sucrose, 1 mM KNO_3_, 1 mM Ca(NO_3_)_2_•4H_2_O, 1.6 mM H_3_BO_3_, 0.8 mM MgSO_4_•7H_2_O) under constant rotation. For every flower, 10 μL pollen suspension was loaded onto a haemocytometer for counting. Pollen with tubes longer than the pollen diameter were considered viable.

### Cytological Analyses

For the cytological and ultrastructural analysis of the developing pollen and tapetum, 3.0 to 3.1 mm flower buds from plants that received a 4-day LTMH or control treatment during 13 to 10 days before anthesis (DBA) were labeled, and anthers were collected on consecutive days after the treatment (see sampling scheme in Supplementary Figure S1A) and fixed in 0.025 M phosphate buffer, pH 7.2, containing 2% glutaraldehyde and 4% paraformaldehyde, for 2 h at room temperature, followed by 2 h post-fixation with 1% osmium tetroxide in water. The samples were then dehydrated in a graded ethanol series and embedded in Spurr's resin. For light microscopy, sections of 1 μm in the median part of the anthers were cut and stained with 0.1% toluidine blue in 1% borax. Sections were viewed and photographed with a Leica DM2500 microscope (Leica Microsystems GmbH, Wetzlar, Germany), equipped with a Leica DFC 420C camera. For electron microscopy, 70 nm sections were cut, post-stained with uranyl acetate and lead citrate according to standard procedures and viewed with a JEOL JEM-1010 (JEOL Ltd., Tokyo, Japan), equipped with a Mega View III camera (Olympus, Soft Imaging System, Münster, Germany).

To describe the progression of pollen development, the number of cells per developmental stage (as described in Supplementary Table S1) was counted in each locule. Pollen was classified as with normal appearance, aberrant in shape and size, and plasmolysed or dead (i.e., no cytoplasm or only remnants of degenerated cytoplasm present). The tapetum development was also staged based on descriptions (Supplementary Table S2). Because tapetum cells of different stages were usually present in one locule simultaneously, the proportions of different stages were scored and the mean value was used. For each time point, the anthers from five to seven flowers were analyzed. In total, 770 locules containing 47,822 developing pollen from the control condition and 758 locules containing 45,016 developing pollen from the 4-day LTMH treatment were analyzed.

To determine the pollen size, fresh pollen at anthesis was viewed and photographed with a Leica DM2500 microscope and Leica DFC 420C camera after staining with 0.1% toluidine blue in 0.1% borax. In total, 2,260 pollen (from 11 flowers) and 1,874 pollen (from 9 flowers) from the control and 4-day LTMH treatments, respectively, were analyzed by using the image analysis software FIJI (https://imagej.nih.gov/ij/). The area of all pollen grains with a round shape was determined.

### Microarray Analysis

On the day after a 4-day LTMH or control treatment, anther cones from 3.6 to 3.7 mm flower buds (corresponding to 9 DBA, see sampling scheme in Supplementary Figure S1A) were collected for total RNA isolation with Trizol reagent (Invitrogen, Thermo Fisher Scientific, Waltham, MA USA), followed by DNA digestion and purification with the RNeasy kit (Qiagen, Venlo, The Netherlands). RNA concentration and quality were checked with a Nanodrop 1000 (ThermoScientific, Thermo Fisher Scientific) and by 1.5% agarose gel electrophoresis. The cDNA was labeled with biotin using the Affymetrix GeneChip WT Terminal Labeling Kit (Affymetrix, Santa Clara, CA, USA) and hybridized to the Affymetrix EUTOM3 tomato exon arrays. The microarray signals were determined using MadMax microarray analysis software (hhtp://madmax.bioinformatics.nl). Robust Multiarray Averaging (RMA) was used for normalization. Original expression data were filtered for average absolute expression value ≥10 for at least one sample type and a log-based IQR ≥0.35, as determined according to (Chockalingam et al., 2016). All remaining transcript data were log-transformed before further analysis to correct for heterogeneity of variance. Principal components analysis was done using ClustVis (Metsalu and Vilo, 2015) with standard settings, that is, with row centring, row scaling with unit variance, and PCA method SVD with imputation. Ranked fold changes were used to determine enrichment of GO-Slim biological process annotations using Panther with false discovery rate correction (http://pantherdb.org/; version 16.0). Genes significantly differently expressed between treatments were identified using FDR q ≤ 0.05 (Benjamini and Hochberg, 1995) and absolute fold change ≥1.5 as cut-offs. Over-representation of GO-slim biological process annotations among up- or downregulated genes was determined using Panther, using the whole tomato genome as a reference, Fisher's Exact test, and FDR correction. A hypergeometric test (http://nemates.org/MA/progs/overlap_stats.html) was applied to test whether the overlap between two subsets of genes was significantly different from expectation. A set of customized pollen and tapetum function associated genes in tomato was generated by fetching the orthologous genes (http://plants.ensembl.org/biomart/martview) to Arabidopsis genes with anther, pollen, and tapetum-related GO annotations, filtering for “all Arabidopsis paralogs are in the GO annotation list” and “two or fewer tomato paralogs”, where applicable (Supplementary Table S3).

### qPCR Analysis

For examination of UPR and tapetum regulatory genes, total RNA isolated for the microarray experiment was used. To examine the expression of reactive oxygen species (ROS) scavenging genes, flower buds received the 4-day LTMH at 13 to 10 days before anthesis (DBA) and control treatments were collected for anther samples at nine time points (Supplementary Figure S1B). Per plant, anthers of multiple flowers were pooled together. Four biological replicates (plants) were used per treatment per time point. Total RNA was extracted with trizol reagent (Invitrogen, Thermo Fisher Scientific). RNA concentration and quality were checked using a NanoDrop 1000 (ThermoScientific, Thermo Fisher Scientific) and the sample was treated with DNase I. Subsequently, cDNA was synthesized using the iScript cDNA synthesis kit (Bio-Rad, Hercules, CA, USA). Primers were designed with Primer3Plus software (primer3plus.com) or Beacon Designer software (PREMIER Biosoft International, Palo Alto CA, USA) (Supplementary Table S4). Primers, SYBRGreen mix (Bio-Rad), and cDNA samples were mixed to a total volume of 25 μL. A 96-well thermocycler (Bio-Rad iCycler) was used for real-time RT-PCR reactions which followed a two-step protocol: 95 °C for 3 min and 40 cycles of 95 °C for 15 s, 60 °C for 45 s. LingRegPCR software (Ruijter et al., 2009) was used to calculate the average amplification efficiency per primer pair, which was combined with Cq values for computation of relative expression levels, which were then normalized against four reference genes, *CAC, SAND, LeEF1*α, and *RPL8*, using GeNorm (Vandesompele et al., 2002).

### Lipid Peroxidation and ROS Scavenging Enzyme Activity Assays

For the lipid peroxidation assay, malondialdehyde (MDA) content was determined with thiobarbituric acid reactive substances assay. A similar set of samples as for qPCR (Supplementary Figure S1B) were ground into a fine powder and homogenized with 1 mL 80% ethanol. After centrifugation at 12,000 rpm for 10 min, 300 μL supernatant was mixed with an equal volume of 0.5% (w/v) 2-thiobarbituric acid in 20% trichloroacetic acid. The mixture was then incubated at 95 °C for 30 min. After cooling and centrifugation at 12,000 rpm for 5 min, the absorbance of the resulted supernatant was measured at 532 nm and 600 nm using a plate reader (SpectraMax 190, Molecular Devices, Sunnyvale, CA, USA). A calibration curve was generated by using 1,1,3,3-Tetraethoxypropane (T9889; Sigma-Aldrich) as a standard. For the ROS scavenging enzyme, samples (Supplementary Figure S1B) were homogenized in 150-μL 0.1 M potassium phosphate buffer (pH 7.0) with 1.0% polyvinylpolypyrrolidone. The extract was centrifuged at 18,000 g for 10 min and the resulting supernatant was stored at −80 °C. Protein concentration was determined as described by Bradford (1976) using bovine serum albumin as a standard. Catalase (CAT, EC 1.11.1.6) activity was determined using the Amplex®Red Catalase Assay Kit (Molecular Probes, Thermo Fisher Scientific) according to the manufacturer's instructions. Assays were performed with 5 μL protein extract and values were corrected for protein concentration. Ascorbate peroxidase (APX, EC 1.11.1.11) activity was determined as described by Nakano and Asada (1981), with minor modifications. A reaction mixture containing 30 μg of protein, 0.5 mL 1 mM ascorbate, and 0.5 mL 0.5 mM H_2_O_2_ was prepared, absorbance at 290 nm was determined after 30 and 240 s, and the difference was used for calculation of relative activity.

### Statistical Analysis

For statistical analysis, pollen viability data were averaged for each plant and logit transformed using the formula PV' = ln((PV+1)/(101-PV)) and relative gene expression data were log transformed. One- or two-way ANOVA followed by Tukey's HSD or LSD *post-hoc* tests were applied to test for statistical significance of differences between treatments. All statistical analyses were performed with SPSS v20 (IBM, NY, USA).

## Results

### Where and When Does LTMH Affect Pollen Development?

**Figure 1:**
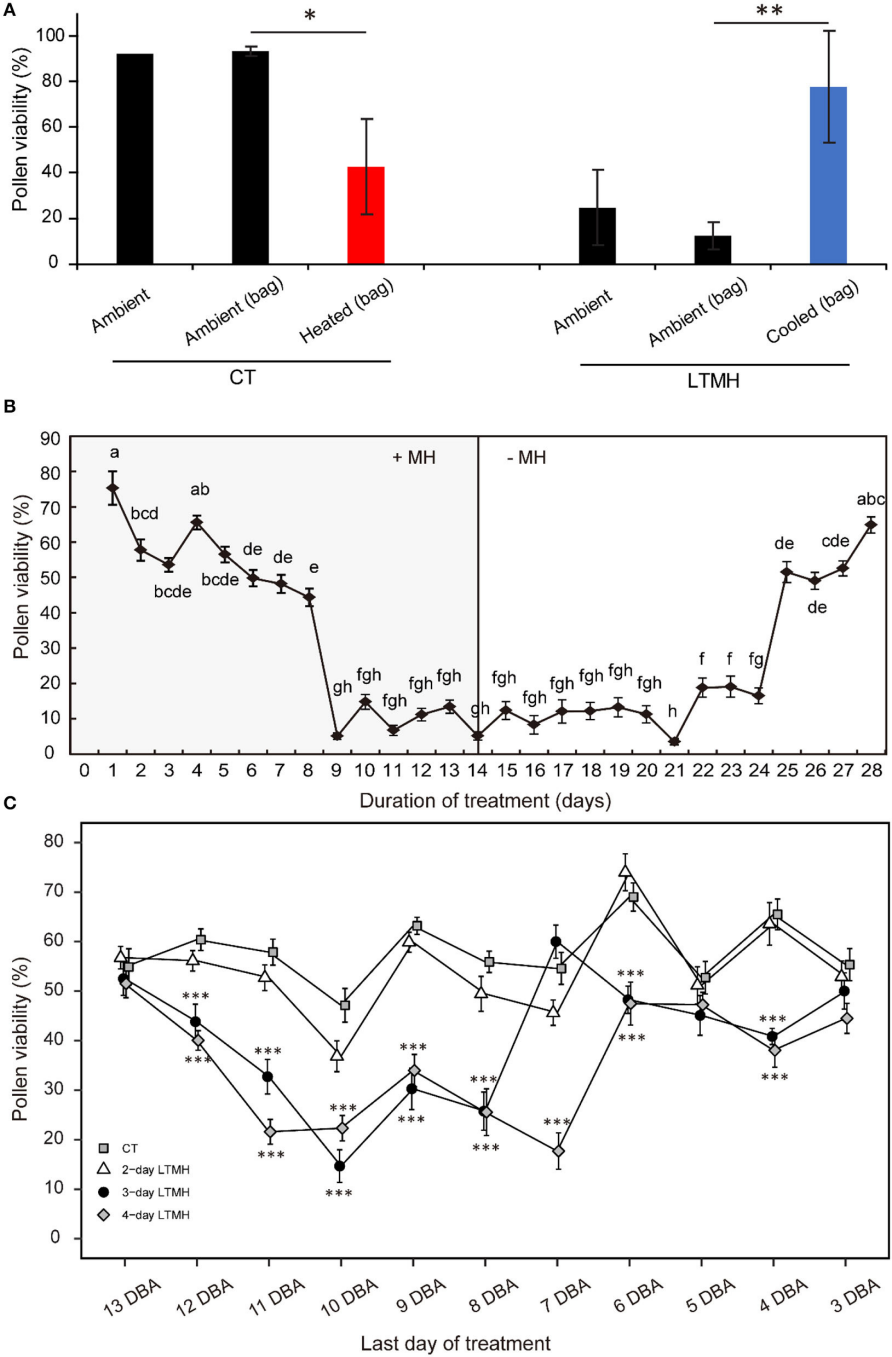
The effects of long-term mild heat location, timing, and duration on viability of mature pollen. **(A)** Local temperature treatments: plants were grown in control (CT) or long-term mild heat (LTMH) conditions, and flower trusses were either exposed to the ambient temperature directly (“ambient”), to the ambient temperature by flushing ambient air through a bag around the truss [“ambient (bag)”], or reduced or increased temperature by flushing air of modified temperature through a bag around the truss [“cooled (bag)” or “heated (bag)”]. Viability of mature pollen (i.e., from freshly opened flowers) was measured 10 to 20 days after the onset of the treatment. Values indicate the mean ± SE (*n* = 2–3 plants, with pools of data from 1 to 3 days, each with at least three flowers per plant). *, significantly different from both controls, one-way ANOVA with LSD *P* ≤ 0.05; **P ≤ 0.01. **(B)** A 4-week stress-and-release experiment, with plants growing in LTMH for 2 weeks, followed by growth in CT for 2 weeks. Viability of mature pollen was determined daily. Values indicate the mean ± SE (*n* = 4 plants, with pools of 2 to 9 flowers analyzed per plant per sampling point). Different letter indicate significant differences between treatments, one-way ANOVA with Tukey *P* ≤ 0.05. **(C)** Effect of LTMH treatments of variable durations and timing on viability of mature pollen. Samples are plotted according to their stage (expressed as days before anthesis; DBA) at the last day of the treatment. The 4-day LTMH treatment that ended at 10 DBA (i.e., flowers treated from 13 until 10 days before they would reach anthesis) was selected for subsequent cytological and gene expression experiments. Values indicate the mean ± SE (*n* = 5 plants, with pools of 2 to 3 flowers analyzed per plant per sampling point). *, significantly different from control at the same sampling point, one-way ANOVA with Tukey *P* ≤ 0.05; **P ≤ 0.01; ***P ≤ 0.001.

To determine whether exposure to long-term mild heat (LTMH) affects pollen development through a local effect on flowers or a systemic effect on the vegetative tissues, we heated single flower trusses of plants that were growing at control temperature (CT; 25/19 °C, day/night) by ~5 °C and, vice versa, cooled flower trusses of plants growing in LTMH (31/25 °C) by ~6 °C, for at least 10 days. In CT conditions, the viability of pollen from the flowers flushed with hot air was significantly lower than that of flowers from the same plant exposed to the ambient CT temperature; in LTMH, the viability of pollen from flowers flushed with cool air was significantly higher than that of flowers on the same plant exposed to the ambient warm LTMH temperature (Figure 1A). This indicates that the LTMH effect on developing pollen is local, at the level of the flower or below.

To determine which pollen developmental stages were sensitive to LTMH, tomato plants were subjected to 2 weeks of LTMH (33/27 °C, day/night), followed by a 2-week release at CT (25/19 °C). Under LTMH, pollen viability of freshly open flowers decreased if they were treated for 3 or more days before reaching anthesis, but a particularly strong reduction was observed when flowers were stressed for at least 9 days before they reached anthesis. When moved out of LTMH, a clear increment in pollen viability was observed in flowers that recovered in control temperature for at least 11 days (Figure 1B). To further determine what period of LTMH exposure was sufficient to induce the pollen damage, plants were exposed to LTMH for 2, 3, or 4 consecutive days only, starting at varying stages of flower development. Slight reductions in the viability of mature pollen were seen if plants were treated with 3- or 4-day MH when treatments ended at 4, 5, or 6 days before anthesis (DBA), while strong reductions were seen if these treatments ended at 8, 9, 10, or 11 DBA. The 2-day LTMH treatment did not significantly decrease pollen viability, irrespective of the flowering stage at which it was applied. Together, these results indicate that the 9-to-11 DBA stage of flower development is highly sensitive to LTMH and that pollen development is compromised if flowers experience at least 3 days of LTMH around this period.

### Pollen Developmental Progression After 4-Day LTMH

**Figure 2:**
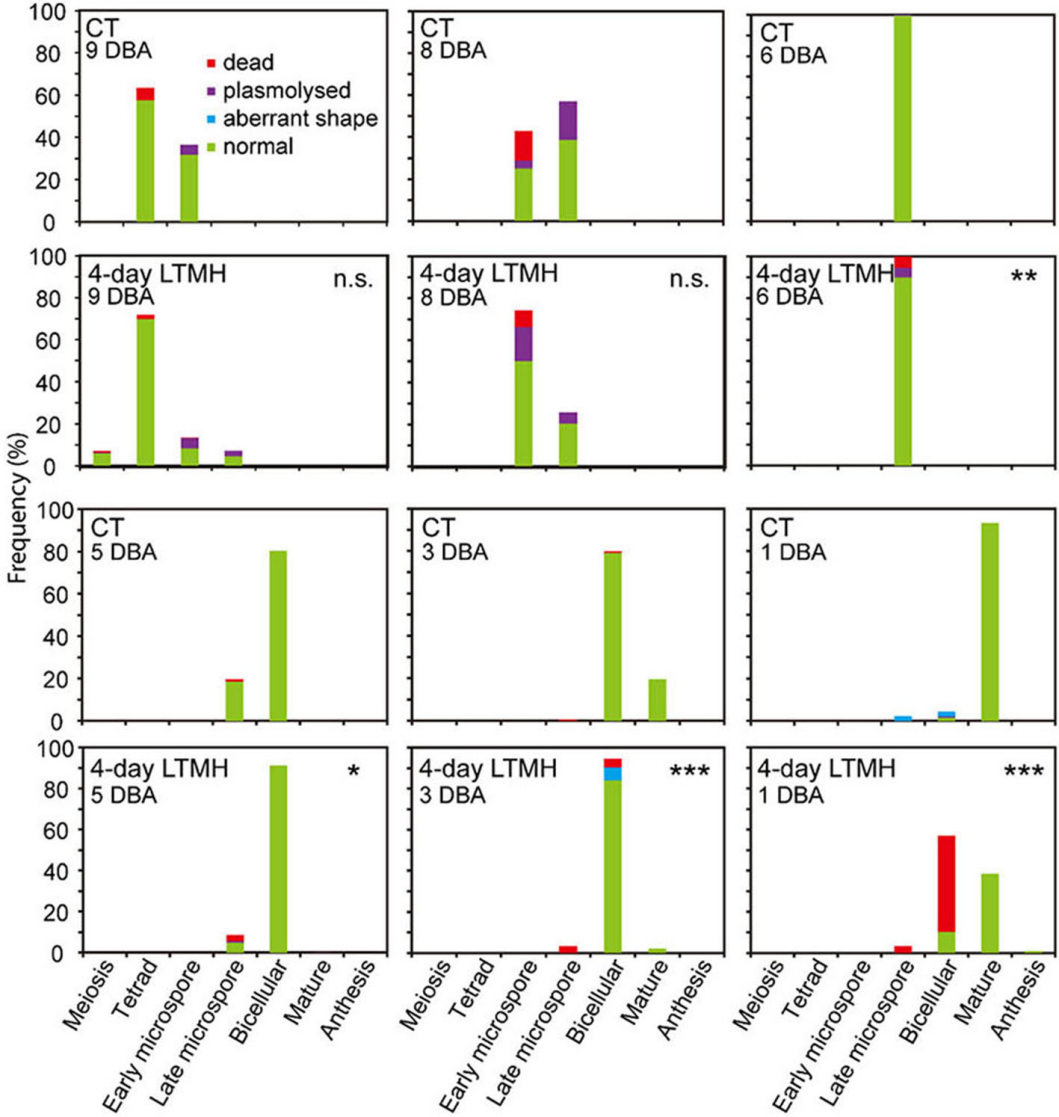
Frequency analysis of pollen development in anthers from control and 4-day long-term mild heat treatment. Long-term mild heat (LTMH) or control temperature (CT) were applied for 4 days, and flowers that were at the 10-DBA stage on the last day of the treatment (i.e., they were treated from 13 DBA to 10 DBA) were labeled and then sampled on subsequent days (at stages of 9, 8, 6, 5, 3, and 1 DBA) for cytological analysis of pollen development (see Supplementary Table S1). For each flower stage per treatment, 100 to 140 locules were analyzed. Developing pollen were assigned to four categories: normal, aberrant shape, plasmolysed, or dead. *, Student's *t*-test, total percentage of normal, living pollen in LTMH treatment significantly less than in CT treatment on the same day *P* < 0.05; **P < 0.01; ***P < 0.001; n.s., not significant.

**Figure 3:**
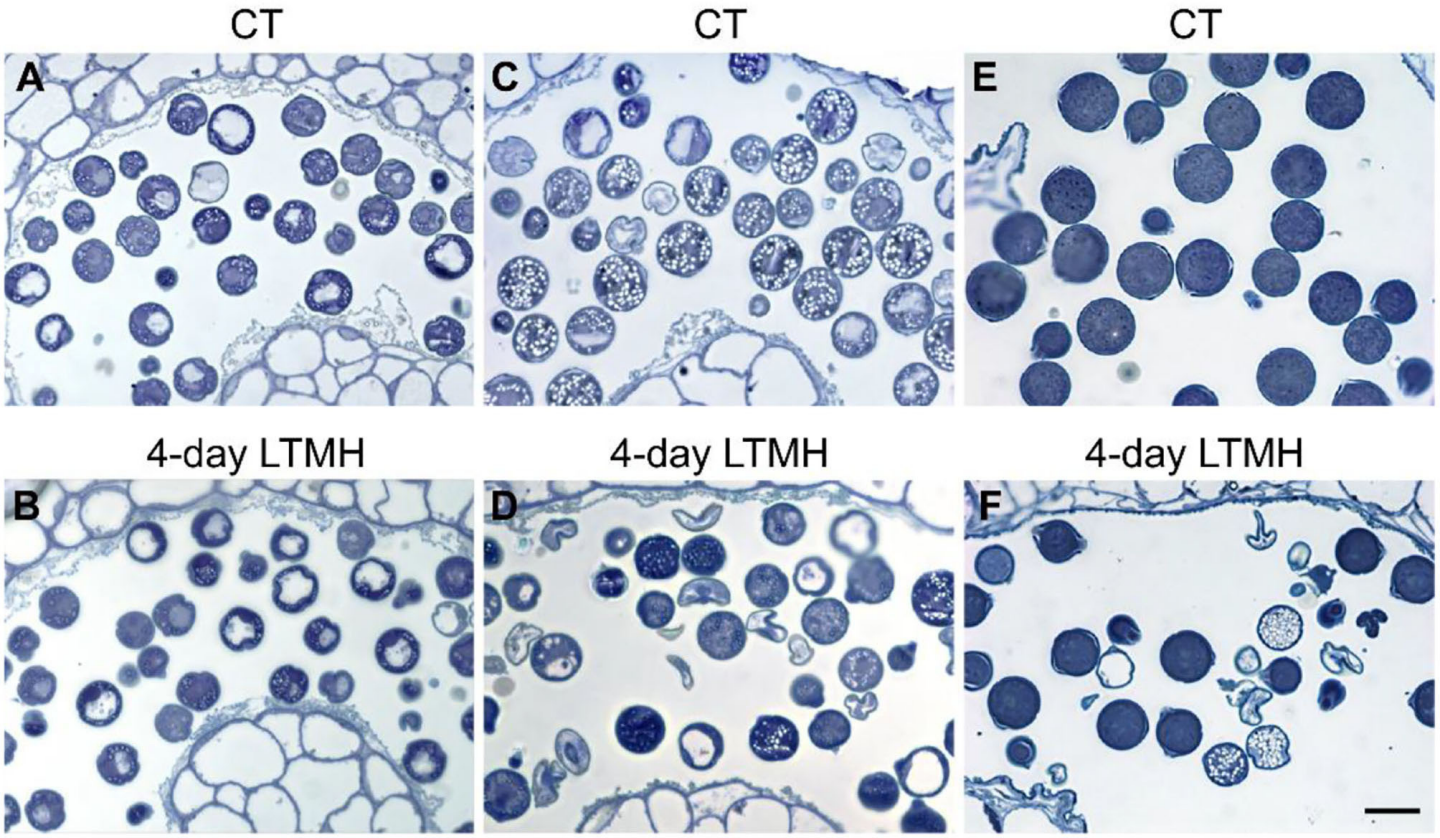
Comparison of pollen cytology after control and 4-day long-term mild heat treatment by light microscopy. **(A,B)** Early bicellular pollen from control (CT) **(A)** and 4-day long-term mild heat treatment (LTMH) **(B)**. **(C,D)** Mature pollen from CT **(C)** and LTMH **(D)**. **(E,F)** Anthesis stage pollen from CT **(E)** and LTMH **(F)**. Bar = 20 μm.

**Figure 4:**
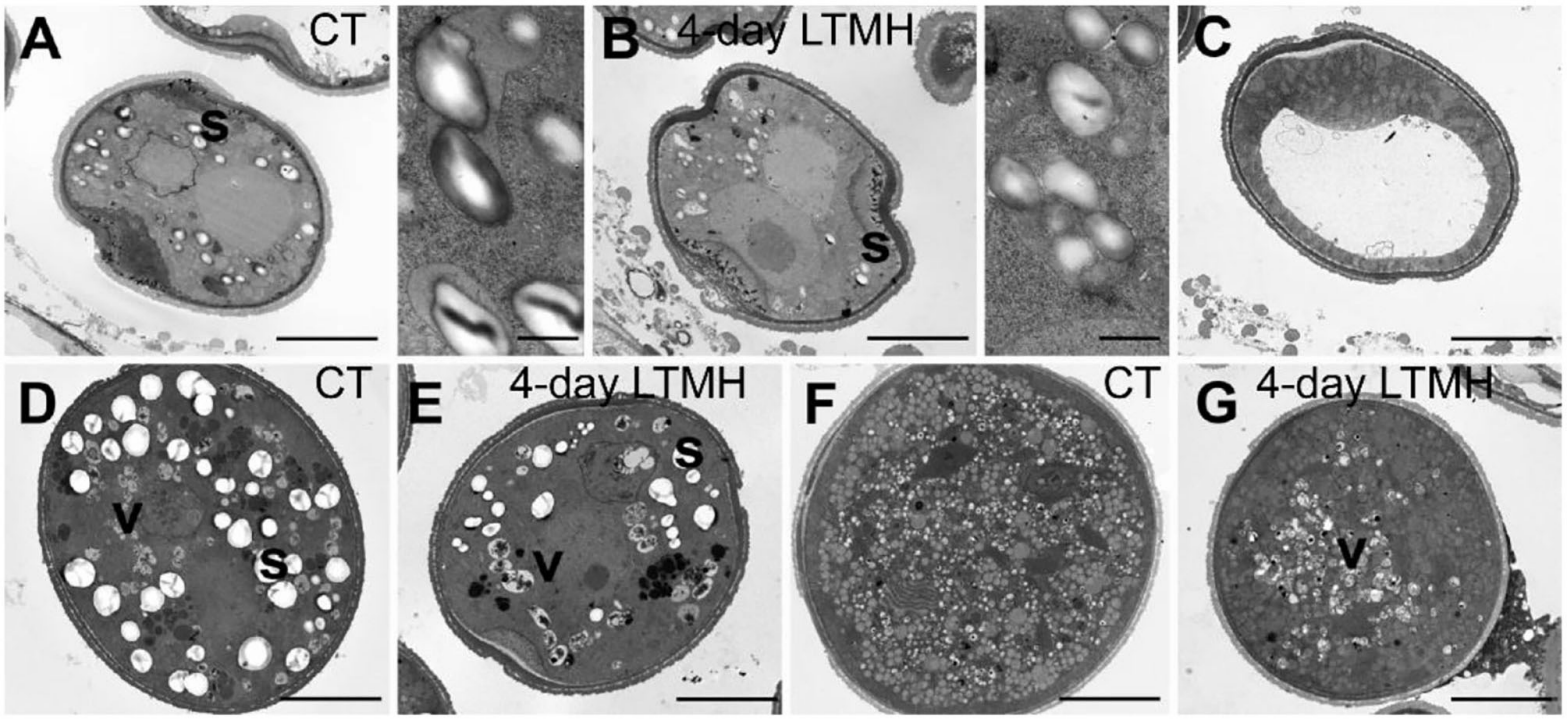
Comparison of pollen ultrastructure between control and 4-day long-term mild heat treatment by electron microscopy. **(A,B)** Early bicellular from control temperature (CT) **(A)** and 4-day long-term mild heat treatment (LTMH) **(B)**, with close-ups of starch granules. **(C)** Second vacuolisation stage, characterized by absence of starch granules, from LTMH. No apparent differences were found relevant to control. **(D,E)** Mature pollen stage from control **(D)** and LTMH **(E)**. **(F,G)** Pollen at anthesis from control **(F)** and LTMH **(G)**. s, starch granule (large, white spherically shaped); v, vacuole (grayish, more or less spherically shaped, with dark debris visible inside). Bar = 5 μm.

**Figure 5:**
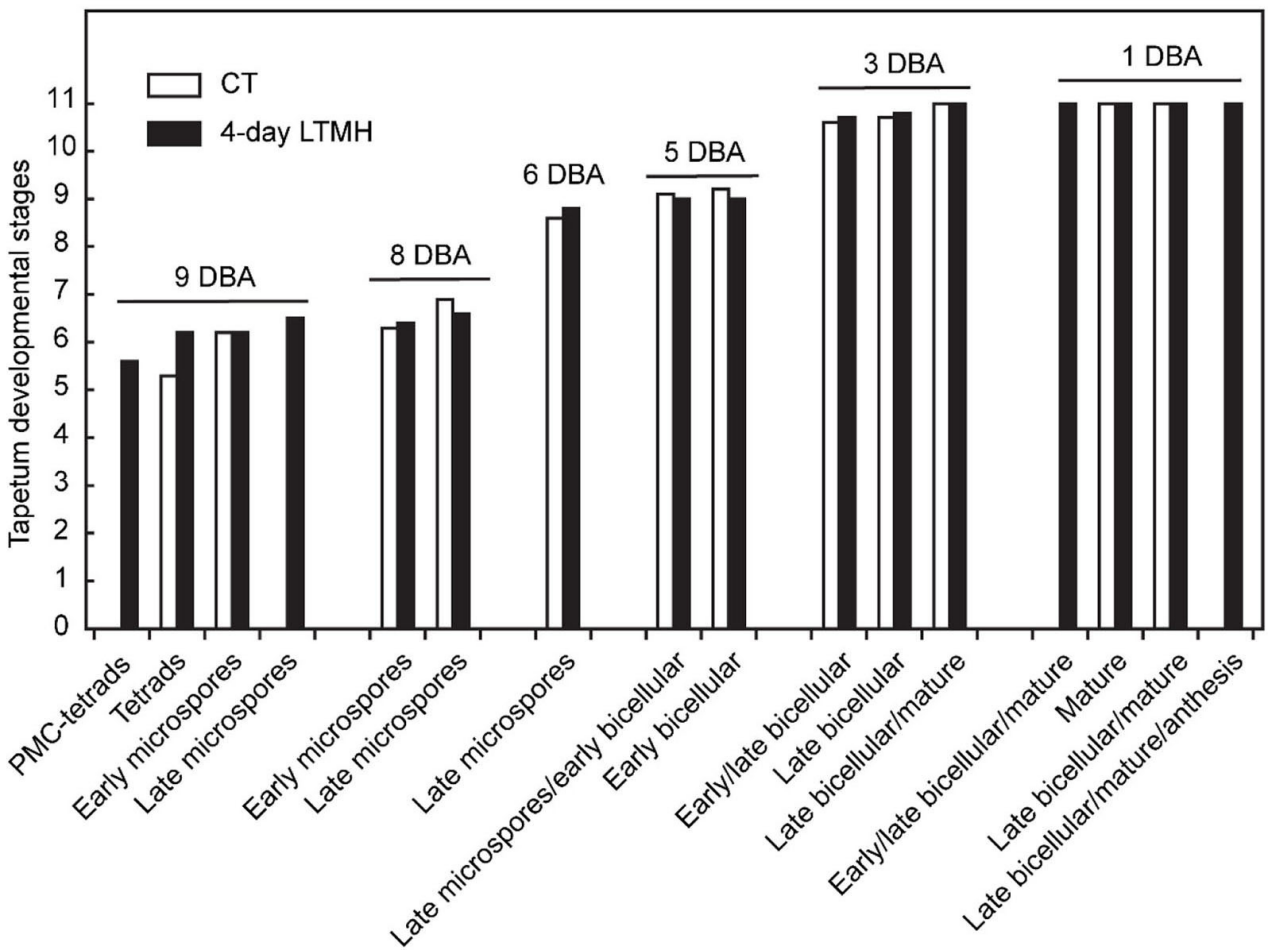
Comparison of tapetum development between control and 4-day long-term mild heat treatment. Long-term mild heat (LTMH) or control temperature (CT) were applied for 4 days, and flowers that were at the 10-DBA stage on the last day of the treatment (i.e., they were treated from 13 DBA to 10 DBA) were labeled and then sampled on subsequent days (at stages of 9, 8, 6, 5, 3, and 1 DBA) for cytological analysis. For each flower stage, average tapetum developmental stage (see Supplementary Table S2) was plotted against pollen developmental stages of the samples (see Supplementary Table S1). For each stage per treatment, 100 to 140 locules were analyzed. pMC, pollen mother cell.

The cytological consequences of a 4-day LTMH treatment ending at 10 DBA (meaning the plants were stressed at 13–10 DBA; Supplementary Figure S1A) on pollen development were determined by labeling flowers at 10 DBA (3.0–3.1 mm buds) and analyzing sectioned anthers on subsequent days. Pollen were mostly at the tetrad stage on the first day after the treatment (i.e., at 9 DBA; Figure 2). No large differences were detected in pollen developmental progression or cell death between the 4-day LTMH and CT treatments in the first 5 days after the treatment (i.e. until flowers were 5 days before anthesis; Figure 2). This included progression through mitosis I at 6 to 5 DBA in both treatment groups. Two days later, at 3 DBA, however, differences between the two treatments became apparent and highly significant in terms of the proportion of normal, living pollen (Figure 2). The bicellular stage started with the accumulation of starch in both, the control and the 4-day LTMH treatment, but this process was more variable in the latter group, where pollen had fewer and smaller starch granules that tended to form clusters (Figures 3A,B, 4A,B). This process was followed by the so-called second vacuolisation, in which the starch disappeared, a large vacuole formed and the size of the pollen gradually increased (Figure 4C). Altogether, pollen proceeded to the late bicellular stage in both treatment groups, but ~10% of the bicellular pollen from the 4-day LTMH became aberrant (i.e., were oval-shaped or had less dense cytoplasm) or died, and few transited into the mature category (Figure 2). Differences were even more clear at 1 DBA and anthesis. With the increase in cytoplasm and disappearance of the large vacuole, numerous new starch granules were formed toward the mature pollen stage. In the 4-day LTMH treatment group, pollen development was less synchronized and again part of the pollen produced fewer and smaller starch grains in comparison with the control (Figures 3C,D, 4D,E). Notably, a large proportion of dead bicellular pollen was observed in the LTMH group at 1 DBA (Figure 2). At the anthesis stage, starch was not present anymore in either, control and the 4-day LTMH treated pollen. No or only small vacuoles were left in control pollen, while in the 4-day LTMH treated pollen, the size and number of vacuoles were more variable and many larger vacuoles were still observed (Figures 4F,G). In addition, anthesis stage pollen from 4-day LTMH were on average smaller than those from CT (Figures 3E,F, 4F,G; Supplementary Figure S2). Contrasting to pollen development, no effect of the 4-day LTMH treatment was observed on the developmental progression of the tapetum relative to time and pollen development (Figure 5; Supplementary Figure S3).

### Transcriptional Responses of Anthers to 4-Day LTMH

**Figure 6:**
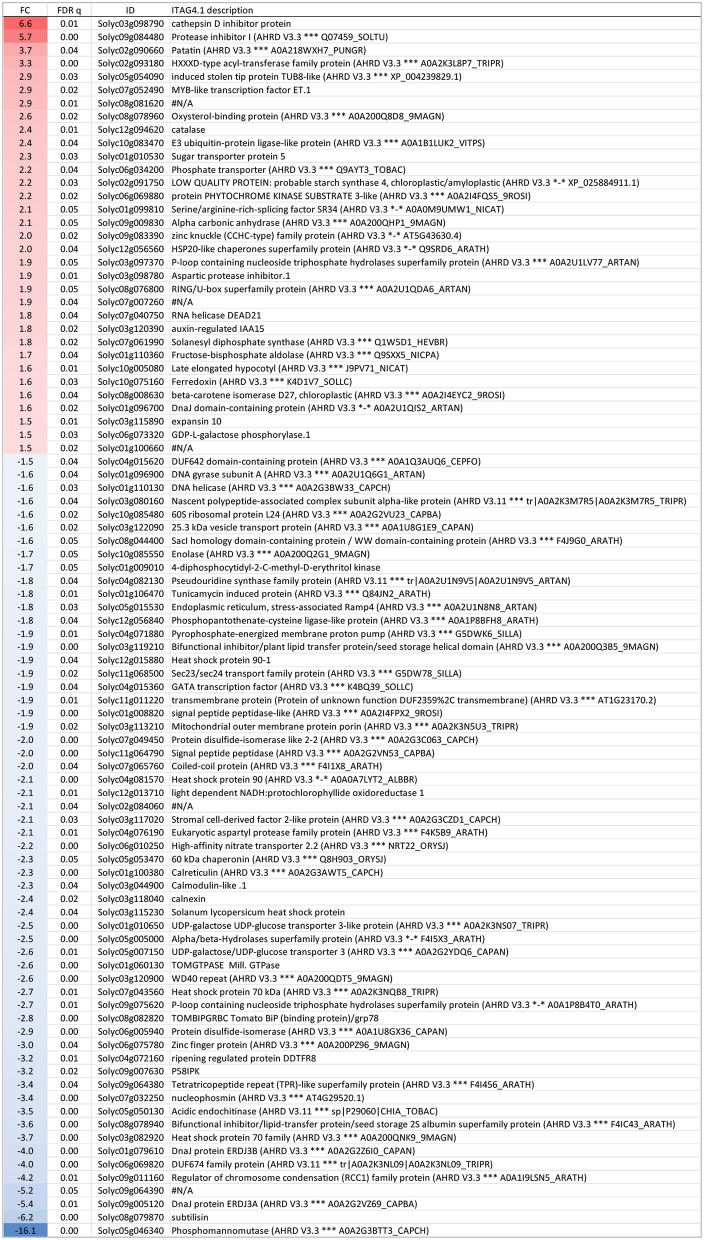
Genes expressed significantly differently (FDR ≤ 0.05 and |fold change| > 1.5) between 4-day long-term mild heat (LTMH) and control temperature (CT) treatments. The first, second, third, and fourth columns are fold change (FC), FDR q value, Solyc gene code, and functional description, respectively. Positive FC value (red label), expressed higher after LTMH than CT; negative FC value (blue label), expressed lower after LTMH than CT.

To get insight into the physiological damage of the 4-day LTMH on male reproductive tissue, developing anthers at the early microspore stage were collected for transcriptome analysis at 3 h into the day after the 4-day treatment (Supplementary Figure S1A; Supplementary Table S5). Principle component analysis showed clear differentiation between LTMH and control samples (Supplementary Figure S4). To obtain a broad overview of modified processes, an enrichment analysis for GO-slim ‘biological process' annotations was done based on ranked fold changes (FC) of all analyzed genes (false discovery rate [FDR] q<0.05). There was a significant bias toward the downregulation of genes related to translation/ribosome assembly, DNA replication/mitosis, protein folding, endoplasmic reticulum unfolded protein response (UPR), and carbohydrate derivative biosynthetic processes (Supplementary Table S6). A slight bias toward upregulation was found for hormone-mediated signaling and flower development-related genes. Among the genes that were significantly differentially expressed between 4-day LTMH and control treatment (|FC|>1.5; FDR<0.05), 33 were found to be upregulated by LTMH and 59 downregulated (Figure 6). No overrepresented GO terms were found among the LTMH-upregulated genes, while, in line with the enrichment test, genes related to protein folding and the endoplasmic reticulum unfolded protein response (UPR) were strongly overrepresented among the LTMH-downregulated genes (Supplementary Table S7). The latter set contained orthologs of the major ER-stress gene BIP and genes related to glycosylation, COPII-coated vesicle budding, and signal peptide processing (see Supplementary Table S8 for qPCR confirmation). A custom set of genes associated with tapetum and pollen development and function was also overrepresented among downregulated genes (Supplementary Table S6; P<0.001). This included orthologs of genes coding for a DNA helicase, a SEC23/24 transport protein, two signal peptide peptidases (SPP), two UDP-galactose/glucose transmembrane transport proteins (UTR3), and tunicamycin-induced protein (TIN1). However, the major genes involved in the regulation of tapetum development were not differentially expressed between CT and the 4-day LTMH (see Supplementary Table S9 for qPCR confirmation). Among the upregulated genes, multiple protease inhibitor genes were present, as well as two ROS-related genes, encoding catalase and vitamin C synthesis protein GDP-L-galactose phosphorylase. qPCR-based expression analysis of a set of ROS scavenging genes confirmed upregulation of *CAT1* during the last day of the 4-day LTMH treatment and several of the following days and showed that APX genes were upregulated during the heat (*APX3*) and several days after the 4-day LTMH treatment (*APX1*) (Supplementary Figure S5). These expression profiles were accompanied by higher catalase and ascorbate peroxidase enzyme activities, while overall lipid peroxidation damage in the anthers was not affected by LTMH (Supplementary Figure S5).

## Discussion

Heatwaves, which consist of multiple consecutive days of relatively high temperature during the warm season, are increasing in frequency and severity globally. In the present work, long-term mild heat (LTMH), as a proxy for heatwaves, negatively affected tomato pollen viability, in line with previous reports (Sato et al., 2000; Pressman et al., 2002; Xu et al., 2017b).

### A General Stress-Sensitive Phase During Early Pollen Development

A stress-and-release experiment indicated that the period around 9 to 11 days before anthesis, covering the meiosis to early-microspore developmental stages, was most sensitive to LTMH, similar to what was reported before in tomato (Sato et al., 2002) and other species (Saini and Aspinall, 1982; Ahmed et al., 1992; Sakata et al., 2000; Porch and Jahn, 2001; Suzuki et al., 2001; Erickson and Markhart, 2002). Furthermore, we found that a short, 3- or 4-day LTMH treatment timed around this period was sufficient to lower pollen viability at anthesis. The same meiosis-to-microspore stage of pollen development was found to be the most susceptible to a short severe heat shock in various species (Iwahori, 1965; Draeger and Moore, 2017; Wang et al., 2017; Begcy et al., 2019), as well as to low temperature and drought in monocots (De Storme and Geelen, 2014). Thus, this early pollen developmental stage seems hypersensitive to physiological disturbance in general.

### Early LTMH Exposure Has a Delayed Effect on Pollen Development

When tracking the development of pollen in flowers that were exposed to a short period of LTMH during the meiosis-to-microspore stage, cytological abnormalities were first detected several days later, during the formation and maturation of the bicellular pollen. Fewer and smaller starch grains were seen during the two successive peaks of starch accumulation, followed by a phase of pollen cell death. Surviving pollen were also smaller, on average, and vacuoles were not fully dissolved at anthesis. Seemingly normal pollen developmental progression after early transient heat, until pollen mitosis or even beyond has also been seen in beans, wheat, and maize (Saini et al., 1984; Sakata et al., 2000; Suzuki et al., 2001; Begcy et al., 2019). This effect is markedly different from that of a short, severe heat shock or absolute high temperatures, which results in immediate defects to meiosis (Draeger and Moore, 2017; Wang et al., 2017; Ning et al., 2021) and abortion of early microspores (Iwahori, 1965; Harsant et al., 2013; Song et al., 2015). Pollen failure due to heat is frequently accompanied by, and ascribed to, mistiming of tapetum degeneration (Parish et al., 2012; De Storme and Geelen, 2014), often late (‘persistent tapetum') in case of severe heat, and premature in case of mild heat (Saini et al., 1984; Ahmed et al., 1992; Sakata et al., 2000; Suzuki et al., 2001; Harsant et al., 2013). We did not detect abnormalities in cytology or persistence of the tapetum cells or mis-regulation of any of the major tapetum developmental regulator genes after early-stage exposure to 4-day LTMH. Endo et al. (2009) described a similar situation in rice under high temperature, with normal developmental progression of the tapetum and normal expression of known major tapetum regulators, while pollen viability was compromised. Also in *Brachypodium*, tapetal defects were not observed at milder heat (Harsant et al., 2013). However, it cannot be excluded that the treatment affected subcellular processes taking place in the tapetum. A role for tapetum in LTMH injury in tomato is suggested by our previous finding that a QTL for pollen LTMH tolerance behaved recessively, and thus acted in a sporophytic tissue (Xu et al., 2017a). Furthermore, we found here that pollen viability was equally affected by the non-overlapping 14–11 DBA and 10–7 DBA LTMH treatments (Figure 1C). Pollen quickly passes through a series of distinct developmental stages in this period, from pollen mother cells to tetrads to free microspores, meaning that either LTMH affects a process that is occurring constitutively in the developing pollen or that LTMH affects a non-pollen tissue that is less dynamic in this period. Indeed, the tapetum may be a more stable cell type in these early stages (Figure 5). In beans, the defects in the tapetum cells were specifically found in the morphology of the endoplasmic reticulum (Suzuki et al., 2001); much more subtle sub-cellular defects may have gone undetected in our analysis and warrant higher-resolution electron-microscopic analysis in the future.

### Protein Folding Stress in the ER

An important question is what damage LTMH does to the young anthers to perturb subsequent pollen development. One of the hallmark effects of high temperature is incorrect folding of proteins, invoking the evolutionarily conserved cytoplasmic heat shock response (HSR) and endoplasmic reticulum stress response (unfolded protein response; UPR), which involve upregulation of the protein folding, modification, repair, and degradation pathways in various cellular compartments (Wang et al., 2004; Li and Howell, 2021). Interestingly, UPR genes constitute a large part of the genes downregulated on the day after the 4-day LTMH treatment, among others those encoding calreticulin, calnexin, BIP, HSP90-ER, protein disulfide-isomerase, and UDP-galactose transporter 3 proteins. Such a transcriptional response points to negative feedback, in which accumulated protective proteins repress the heat stress response, even to below control levels directly after the stress is released, as has been seen before (Bita et al., 2011; Liu et al., 2012; Bonnot and Nagel, 2021). So, while the earliest response of anthers to mild and severe heat mainly involves HSR-related genes (Bita et al., 2011; Fragkostefanakis et al., 2016b), the long-term response to LTMH seems to involve modulation of UPR-related gene expression. Interestingly, recent studies indicated that an intact UPR is essential for pollen development under very mildly elevated temperatures (Deng et al., 2016; Fragkostefanakis et al., 2016a; Feldeverd et al., 2020; Yamamoto et al., 2020; Lu et al., 2021). It has been suggested that the ER in the tapetum is under constitutive strain already under normal conditions, which could explain the high sensitivity of this tissue to additional stress (Singh et al., 2021). Therefore, it is worthwhile to further investigate the role of tapetum ER stress and its protection in heat-mediated pollen sterility.

### Oxidative Stress

Reactive oxygen species (ROS) accumulation is considered secondary stress, often occurring as a result of exposure to a primary, external stress factor. ROS levels are finely tuned to maintain proper cellular homeostasis and are regulated in a developmental, stress, and feedback-dependent manner (Huang et al., 2019). During male reproductive development, deviations in ROS regulation lead to tapetal dysfunction and pollen abortion (Hu et al., 2011; Luo et al., 2013; Xie et al., 2014; Yi et al., 2016). In our study, the expression of *APX* genes was found to be induced during the LTMH treatment, in accordance with a previous report on early LTMH responses (Bita et al., 2011). Furthermore, *CAT1* and *APX3* expressions were still high in the following days. This pattern corresponded with increases in the respective enzyme activities. The upregulation is likely due to feedback regulation by elevated ROS levels (Rizhsky et al., 2004), but as no overall oxidative damage in the form of lipid peroxidation was detected, the increase in scavenger activity seems to have largely maintained ROS balance upon LTMH. This differentiates LTMH from severe heat, where oxidative damage is often detected, possibly because the activity of various scavengers is reduced at high absolute temperature (Panchuk et al., 2002; Djanaguiraman et al., 2014, 2018; Zhao et al., 2018). It is still possible that, under LTMH conditions, ROS caused damage in a specific subset of cells or tissues of the anther which remained undetected. In support of this notion, the tolerance of pollen development to LTMH was somewhat enhanced by overexpression of a glutaredoxin S17 (Müller et al., 2016) and by increasing endogenous flavonol levels (Rutley et al., 2021).

### Carbohydrate Limitation and Partitioning

Developing microspores/pollen cells act as a sink and are thought to compete for limiting resources available in the anther locules, making them sensitive to disturbances in supply, use, and partitioning of nutrients (García et al., 2017). Starch and soluble sugar levels are finely regulated during pollen development: in plants grown under optimal conditions, starch accumulates after pollen mitosis I, followed by a break-down to form soluble sugars at the anthesis stage. These simple sugars then serve as the energy source for imminent pollen germination. In tomato, treatment with LTMH during the whole period of flower development significantly decreased the starch concentration in bicellular pollen and consequently, reduced soluble sugar content at anthesis (Pressman et al., 2002; Firon et al., 2006; Sato et al., 2006). We observed two distinct instances of starch accumulation in bicellular pollen, interrupted by the second vacuolisation and the 4-day LTMH treatment resulted in fewer and smaller starch granules in both instances. Reduced carbohydrate availability was also suggested by the significant bias toward the downregulation of the gene set associated with carbon derivative biosynthetic processes. As carbohydrates also act as osmolytes in dehydrating pollen (Firon et al., 2012), the LTMH-induced reduction of pollen volume at the final stage might well be the result of a low soluble sugar content. Questions remain as to the step in carbohydrate transport, partitioning, or metabolism affected by LTMH and whether the observed deviations are causal to pollen failure or rather a symptom thereof. The fact that the reduced pollen viability phenotype correlated to the direct surrounding temperature of the flower, and not to that of the rest of the plant, indicates that source activity and phloem loading are not limiting factors for pollen development under LTMH. Carbohydrate metabolism and transfer from the anther and tapetum to the developing pollen may be affected by LTMH. As we did not find downregulation of genes involved in these processes after LTMH, such as those for invertases and sucrose synthases (De Storme and Geelen, 2014), this would need to have occurred during the heat or involve post-transcriptional regulation. Alternatively, it has been hypothesized that carbohydrates are titrated away from developmental processes toward the mounting of an acclimating response (Rieu et al., 2017). Also, we found that several protease inhibitors were upregulated after LTMH, which may suggest extra investment in defense against pathogens and insects (Mosolov and Valueva, 2005).

In conclusion, our results show that LTMH affects male fertility through a direct, local effect on flowers and reveal fundamental differences between LTMH and severe heat-induced pollen failure, in terms of timing of pollen developmental deviations, severity of tapetum defects, and cellular damage. This suggests that multiple tolerance traits might be needed to improve plant reproductive performance in high-temperature conditions.

## Data Availability Statement

The raw microarray data generated in this study are available in the NCBI GEO repository under accession number GSE180168 and the processed data can be found in the supplemental datasets provided.

## Author Contributions

JX, SJ, and IR conceived and designed the study and wrote the manuscript. JX, SJ, MW-A, PG, and MJ performed the research. JX, SJ, MW-A, and IR analyzed and interpreted the data. All authors contributed to the article and approved the submitted version.

## Funding

This work was supported by the China Scholarship Council (CSC Grant Number 201207565002, to JX), the Dutch Research Council (NWO Grant Number 867.15.011, to IR), and the Dutch Topsector Horticulture and Starting Materials (TKI TU grant number TU-2018-012, to IR).

## Conflict of Interest

The authors declare that the research was conducted in the absence of any commercial or financial relationships that could be construed as a potential conflict of interest.

## Publisher's Note

All claims expressed in this article are solely those of the authors and do not necessarily represent those of their affiliated organizations, or those of the publisher, the editors and the reviewers. Any product that may be evaluated in this article, or claim that may be made by its manufacturer, is not guaranteed or endorsed by the publisher.
