# Supplementary material for: Long-Term Mild Heat Causes Post-Mitotic Pollen Abortion Through a Local Effect on Flowers
Source: Front Plant Sci. 2022 Jul 11;13:925754. doi: 10.3389/fpls.2022.925754 (PMC9310381; doi:10.3389/fpls.2022.925754)
Supplement: Supplementary file 4 [file Data_Sheet_1.DOCX]

**Supplementary Tables and Figures**

Table S1. Definition of pollen developmental stages.

| Stage | Description |
| --- | --- |
| Pre-meiotic | Differentiation of pollen mother cells (PMCs) and tapetum. The pollen mother cells are in an angular shape and their nucleus with nucleolus and small vacuoles are visible. |
| Meiosis | Meiotic division until anaphase II. PMCs become less angular and tend to be round. Callose deposition around PMCs. Separation of tapetum starts. |
| Tetrads | From the end of meiotic division, telophase II and cytokinesis until the start of callose dissolution. Callose deposition between microspores and formation of tetrads. |
| Early Microspore | Callose degradation leads to the release of free microspores in which apertures are visible. Microspore cell wall is thickening. Irregularly shaped microspores slowly turn spherical. |
| Late microspore | Microspores are spherical and larger. Pores become clearly visible. Microspores undergo vacuolisation, forming numerous (>5) vacuoles that will merge, forming one large vacuole. |
| Bicellular | Mitotic division gives rise to two cells. At the early bicellular stage, the generative cell is appressed to the pollen cell wall and smaller than the vegetative nucleus. The large vacuole disappears and smaller and irregular vacuoles are visible. Accumulation of starch and intine thickening at apertures happens. The generative cell is free in the cytoplasm. During the following second vacuolisation, a new large vacuole appears and starch granules disappear. |
| Mature | Mature pollen become large spherical grains. The vacuole disappears and many starch granules are visible around the nucleus. Numerous lipid droplets and small vacuoles can be present. |
| Anthesis | Spherical pollen, starch has disappeared. Black inclusions in the cytoplasm, lipid droplets are prominent. Faint nucleolus. |

Table S2. Definition of tapetum developmental stages.

| Stage | Description |
| --- | --- |
| 1 | Differentiation pollen mother cells and tapetum. Small angular cells with a large nucleus, as well as small and large vacuoles. |
| 2 | The tapetal cells become bicellular, elongated and contain small vacuoles. |
| 3 | The tapetum starts to separate from pollen mother cells. Cell wall at locule side of tapetal cells is dense stained. |
| 4 | Larger vacuoles with dense stained deposits. Outer walls at the locule side appear to be thicker and more densely stained. |
| 5 | The tapetal cells become irregular shaped and cell walls are weakly stained. Sometimes, bluish lines at the locule side visible. Few small round orbicules deposited on the walls at the locule side and in between the tapetum cells. Some small grey droplets in the cytoplasm are visible. Hardly any nucleoli visible in the nuclei. |
| 6 | Large amounts of round orbicules deposited on the walls at the side of the locule and in between tapetum cells. In vacuoles and cytoplasm, larger grey droplets are observed. Hardly any nucleoli visible in the nuclei. |
| 7 | Tapetum starts to degenerate. The signs of degradation are solid dense stained cytoplasm, nucleus dissolving, vacuoles aggregate and become larger. Cells show strong plasmolysis. |
| 8 | The tapetal cells are degrading and cytoplasm becomes less dense stained. Large grey-brown spots are clearly visible. A small amount of remnants of densely stained cytoplasm are still present. |
| 9 | All tapetal cells show remnants of degraded cytoplasm. Nuclei are disintegrated and Large grey-brown spots are visible. |
| 10 | Only packed cell walls left with remnants of large grey-brown spots and dense deposits, no remnants of cytoplasm. |
| 11 | Packed cell walls with hardly any remnants of grey-brown spots and dense deposits. |

Table S3. A) GO terms used to extract pollen, tapetum and anther-related genes of Arabidopsis from the TAIR10 annotation. B) Tomato orthologs of Arabidopsis pollen and tapetum and anther-related genes extracted from TAIR10 using GO terms listed in Table S3A.

SEE EXCEL SHEET

Table S4. Primers used for qRT-PCR in this study.

| Gene | Solyc code | Forward primer (5'-->3') | Reverse primer (5'-->3') |
| --- | --- | --- | --- |
| *UPR-related genes* | | | |
| *BiP* | Solyc08g082820 | TTGATGCCCGTAACGCTTTG | TCAAGGGCTTCTTTCGTTGC |
| *BiP* | Solyc03g082920 | AACCGCCACAAAAGAAGCAC | TTGCACACTGCCTCAACTTC |
| *CNX* | Solyc03g118040 | AAGATCTGGGCGCAATATGC | ATCGCCCATCAAATGCTTCG |
| *CRT* | Solyc01g100380 | TGCCAAGTCAATTGCAGAGG | TCATCGGCATCAGATTCAGC |
| *ERdj3b* | Solyc01g079610 | ATGCCCCAACGTGAAGTTTG | TTCCTGGCCATCTTGCATTC |
| *HSP70* | Solyc07g043560 | AACACCGGTGAAAGATGCTG | TCATTCACAAGCGCCAACAC |
| *PDI* | Solyc06g005940 | ACGCAACCGCAAATGATCTC | TGCCACGATTCTTCTCGATG |
| *PDI* | Solyc07g049450 | TGTTGTTAGCCGTTGCAGAG | AACCTCCAACACCAACATGC |
| *Ramp4* | Solyc05g015530 | GTGACGCGTTTTTCTTGTGC | TTGTTCCTCCTTCCCTTTGC |
| *SDF2* | Solyc03g117020 | ATCCGCAAAGAGCTTCATCG | TGCACCGGCTTGATTCAATG |
| *SPP* | Solyc11g064790 | TTTGTTTCCCACAGCGGATG | GCCAATGCGACAAAAATGCC |
| *SPP* | Solyc01g008820 | AGTGCATTTTCGGGATACGC | TTCCAAATGCAGTGCACAGC |
| *UTR3* | Solyc05g007150 | TGTTGCTGGTGGTGTTTCAC | AGTGGAGCATTTGGATGTGC |
| *UTR3* | Solyc01g010650 | AGGGCAAAACACCGAAGTTG | TCGGCGCTAACACTTTGATC |
| *Tapetum-regulatory genes* | | | |
| *AMS1* | Solyc08g062780 | AGCAAATGGAGCCACAAGTG | AGCCCAATGAGTTCAAAGCC |
| *bHLH89* | Solyc01g081090 | TCTCCAACATGTTGCTGGTG | TGTCTGCATGTTCCTTGTCC |
| *bHLH89* | Solyc01g081100 | TGTTCGGATTGTCGACGATG | CACCAATAAGTCCACCAGCAAC |
| *MS1* | Solyc04g008420 | ATGGTCACCTAAACGCGTTG | ATAAGCACGAGCAGCATCAC |
| *MS2* | Solyc03g051960 | AGCTGACATGGTTGTGAACG | ACAGCAGATGAAGCAACCTG |
| *MS10* | Solyc02g079810 | TTGGCCCAACTAAGCTTTGG | TTTAGAGGCAGTGGCACTGG |
| *TDF1* | Solyc03g059200 | TTGCTCAGGTCTTTGCTGAG | TGTTGCAAGGGATGATGTGG |
| *ROS-related genes* | | | |
| *APX1* | Solyc06g005160 | AGATGCTGATTGGAAGAG | GGAAGAGTGTCACAATATC |
| *APX2* | Solyc06g005150 | CTTCAGATCAACTACTATGG | GACGAAGCATAATAGGAG |
| *APX3* | Solyc09g007270 | CCGATTATGCTGAGATTAG | TAGGATAGGATTGGGAAC |
| *APX4* | Solyc01g111510 | CTCACGGTGCTAATAATG | CCAGGGACAAATTCAATAG |
| *APX5* | Solyc02g083620 | TCAGTGTCCCGTATTATC | CCTCCAGTCTTAGTTGTA |
| *CAT1* | Solyc12g094620 | GGAGGATACTTGATTTCTTC | CAGTGGAACTTCACATAATG |
| *CAT2* | Solyc02g082760 | GGTATTCCACAAGATTACAG | AATAGAGTCATAGAGGTCCT |
| *CAT3* | Solyc04g082460 | CCAAGGATCTCTATGATTCA | CGATGTTCCTATTCAATACC |
| *CSD1* | Solyc01g067740 | AACAGGACCACATTACAATC | AATCTGCTTGTCGGTAATAG |
| *CSD2* | Solyc06g049080 | GCATCATCAGACTTACATC | GGCAAGGTTATTCCAGAA |
| *CSD3* | Solyc03g062890 | AATGGAGAAAGATGTAGGG | GACCAATTATTCTTCCTCTC |
| *GPX4* | Solyc09g064850 | AAGAGGACAGTAGAAGAAG | GGACGGATGGTATAGTTC |
| *GPX8* | Solyc12g056240 | CCACAATCTGTCTATGAC | CCGTGTAGTTTGAGTTAG |
| *Reference genes* | | | |
| *SAND* | Solyc03g115810 | TTGCTTGGAGGAACAGACG | GCAAACAGAACCCCTGAATC |
| *CAC* | Solyc08g006960 Solyc06g061150 | CCTCCGTTGTGATGTAACTGG | ATTGGTGGAAAGTAACATCATCG |
| *LeEF1α* | Solyc11g069700  Solyc06g009970  Solyc06g005060  Solyc06g009960 | TGATCAAGCCTGGTATGGTTGT | CTGGGTCATCCTTGGAGTT |
| *RPL8* | Solyc10g006580 | GGTGTTCTGGTGATTACGCCATTG | CCAGCAACCTGACCAATCATAGC |

Table S5. Gene expression analysis of CT and 4-day LTMH treatments.

SEE EXCEL SHEET

Table S6. GO-Slim Biological Process gene set enrichment analysis of the transcriptomic response to 4-day LTMH.


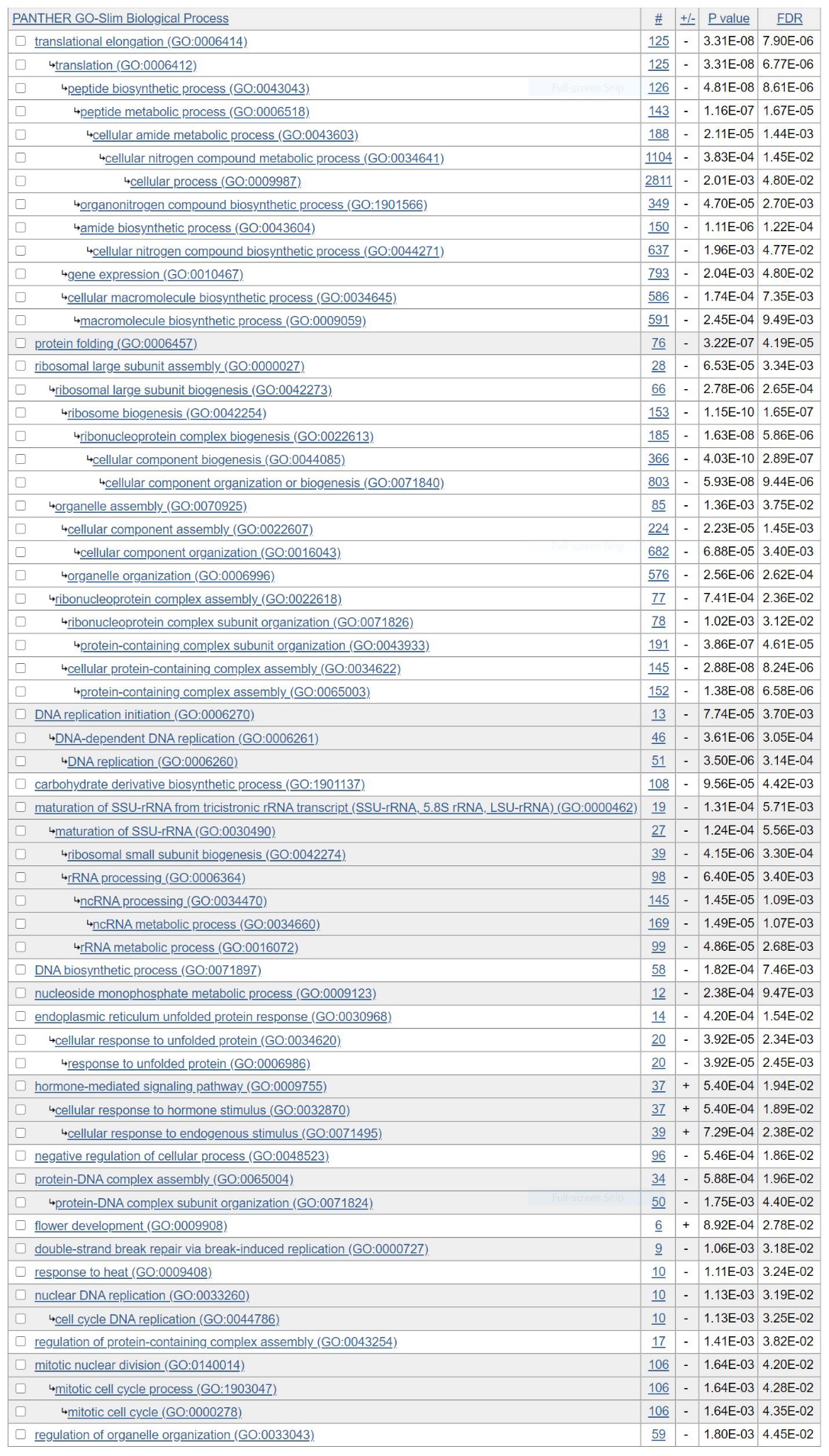


Table S7. GO-slim Biological Process overrepresentation analysis among 4-day LTMH vs CT differentially expressed genes.


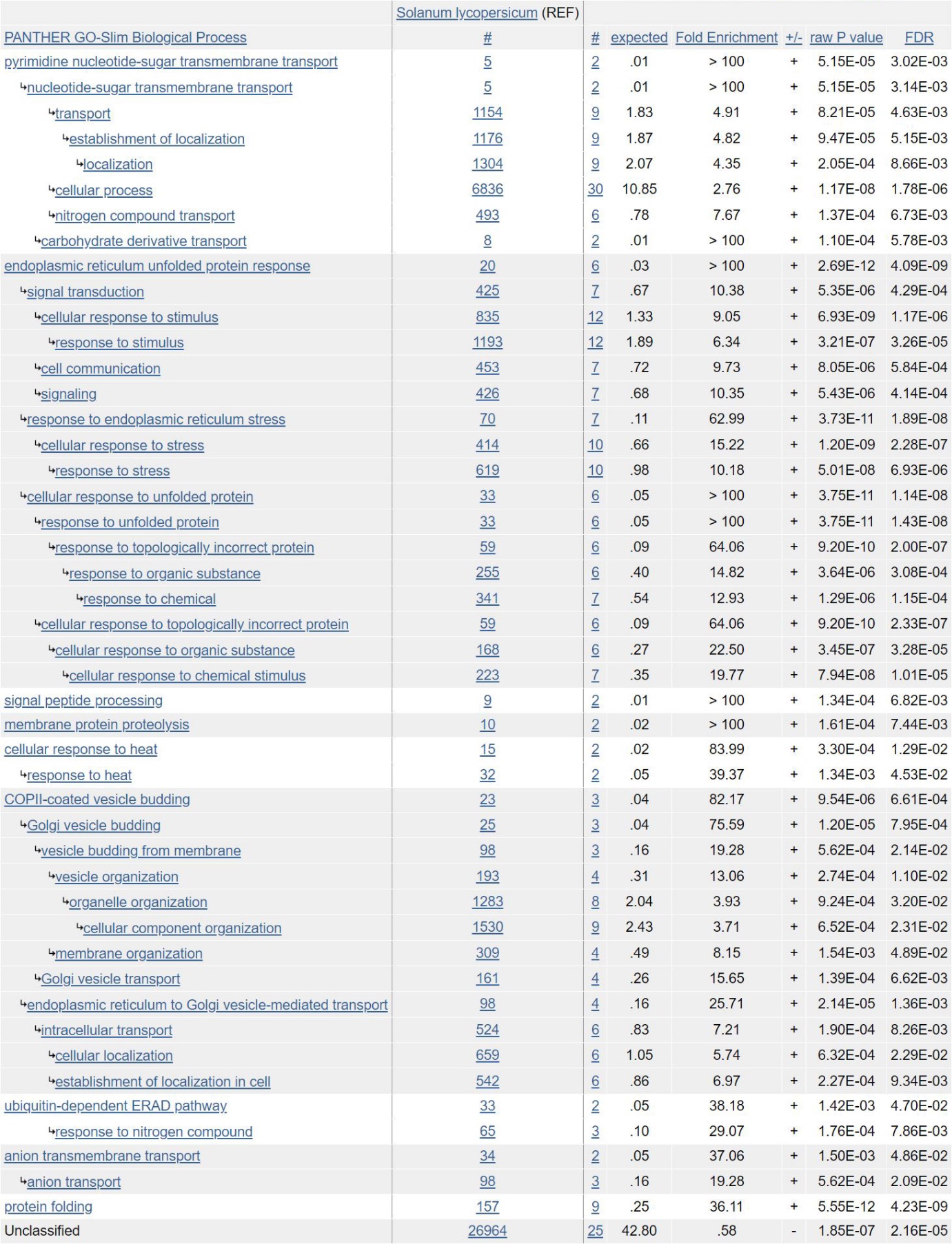


Table S8. qPCR confirmation of expression of UPR-related genes differentially expressed after LTMH treatment according to the microarray analysis.

| Gene | Solyc code | Microarray FC^1^ | Microarray FDR^2^ | qPCR FC^3^ | qPCR P^4^ |
| --- | --- | --- | --- | --- | --- |
| ERdj3b | Solyc01g079610 | -4.0 | ** | -2.8 | *** |
| BiP | Solyc03g082920 | -3.7 | ** | -3.2 | ** |
| PDI | Solyc06g005940 | -2.9 | ** | -3.2 | *** |
| BiP | Solyc08g082820 | -2.8 | ** | -3.2 | ** |
| HSP70 | Solyc07g043560 | -2.7 | * | -1.6 |  |
| UTR3 | Solyc05g007150 | -2.6 | * | -2.6 | ** |
| UTR3 | Solyc01g010650 | -2.5 | ** | -1.1 |  |
| CNX | Solyc03g118040 | -2.4 | * | -2.1 |  |
| CRT | Solyc01g100380 | -2.3 | ** | -2.5 | *** |
| SDF2 | Solyc03g117020 | -2.1 | * | -1.9 | * |
| SPP | Solyc11g064790 | -2.0 | ** | -1.7 | ** |
| PDI | Solyc07g049450 | -2.0 | ** | -2.0 | ** |
| SPP | Solyc01g008820 | -1.9 | ** | -1.6 | ** |
| Ramp4 | Solyc05g015530 | -1.8 | * | 2.4 |  |

^1^ Data extracted from Table S5. Fold change (FC) is maximum/minimum value, with a positive value indicating up- and a negative value indicating downregulation in LTMH vs CT.

^2^ Data extracted from Table S5. * FDR q<0.05; ** <0.01; *** <0.001.

^3^ Data from qPCR analysis, with FC values as explained under (1)

^4^ Student’s t-test. * P<0.05; ** <0.01; *** <0.001.

Table S9. qPCR confirmation of expression of major tapetum-regulatory genes.

| Gene | Solyc code | Microarray | | qPCR | |
| --- | --- | --- | --- | --- | --- |
|  |  | **FC^1^** | **FDR^2^** | **FC^3^** | **P^4^** |
| AMS1 | Solyc08g062780 | 1.2 | ns | 2.0 | ns |
| bHLH89 | Solyc01g081090 | -1.1 | ns | 1.4 | ns |
| bHLH89 | Solyc01g081100 | 1.1 | ns | 1.6 | ns |
| MS1 | Solyc04g008420 | 1.1 | ns | 1.5 | ns |
| MS10 | Solyc02g079810 | 1.7 | ns | 3.0 | * |
| MS2 | Solyc03g051960 | -1.7 | ns | -1.2 | ns |
| TDF1 | Solyc03g059200 | -4.5 | ns | -3.3 | ns |

^1^ Data extracted from Table S5. Fold change (FC) is maximum/minimum value, with a positive value indicating up- and a negative value indicating downregulation in LTMH vs CT.

^2^ Data extracted from Table S5. * FDR q<0.05; ** <0.01; *** <0.001; ns, not significant.

^3^ Data from qPCR analysis, with FC values as explained under (1)

^4^ Student’s t-test. * P<0.05; ** <0.01; *** <0.001; ns, not significant.

**
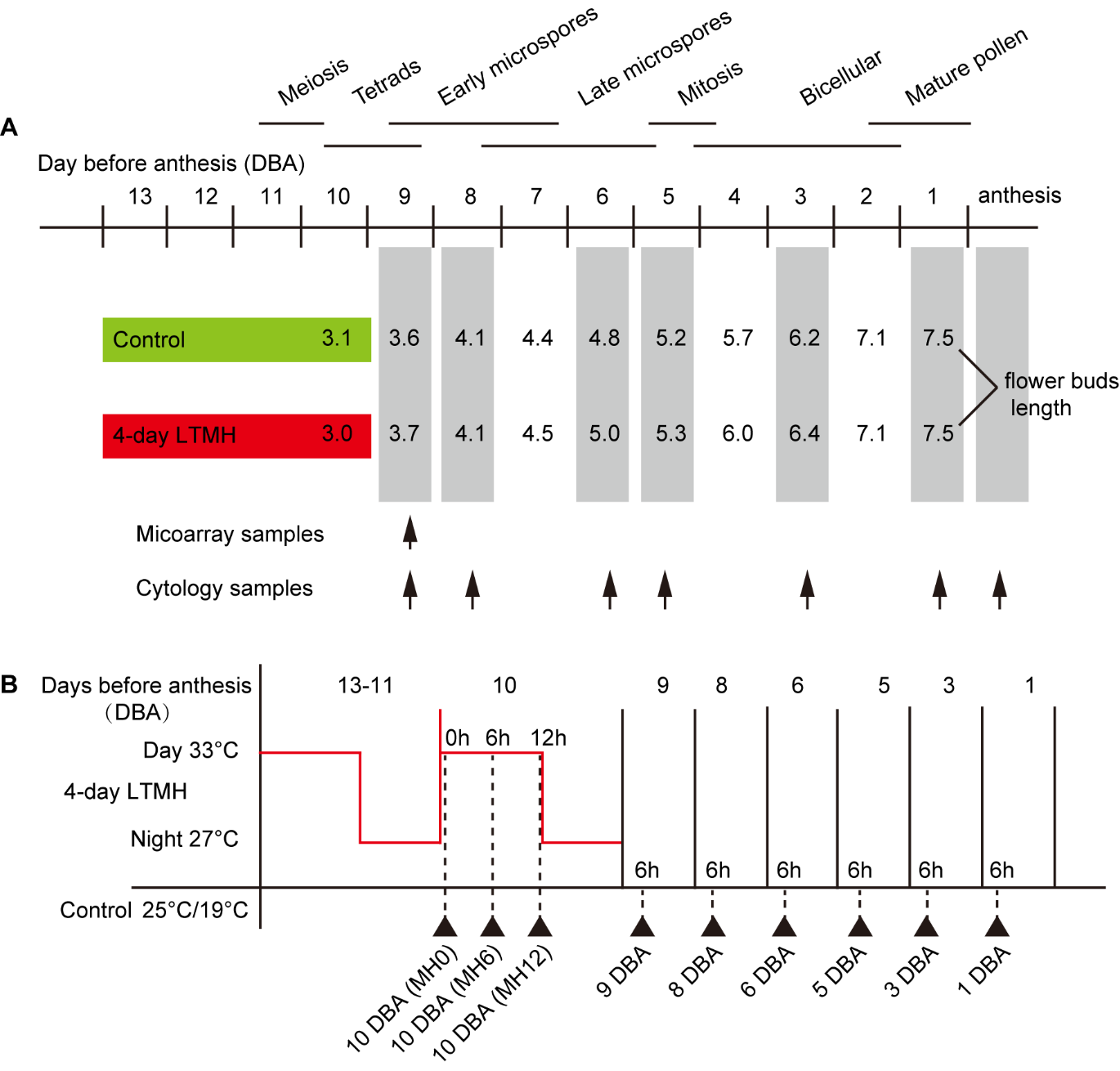
**

**Fig S1. Experimental setup for cytology, microarray and qPCR analyses.** A) Sampling scheme for cytology and microarray experiments. The 4-day long-term mild heat (LTMH) was imposed from 13 days before anthesis (DBA) until the end of 10 DBA. From our observation, tomato flower buds length is about 3.0-3.1 mm at DBA and therefore, ten flower buds of such length were tagged for CT and LTMH treatment eachs. The length of the ten reference flowers (which was not affected by LTMH treatment) was used to identify similarly aged flowers for anther sample collection during and after the LTMH treatment. For cytological observation, samples were collected at 7 time points (9, 8, 6, 5, 3, 1, and 0 DBA), and for microarray analysis, anthers were collected on 9 DBA (3 hours after the start of the light period). B) Sampling scheme for the qPCR experiment. Anthers were taken during the last day of the 4-day LTMH treatment (i.e. on 10 DBA, at three time points after the start of the light period) and in the middle of subsequent days.


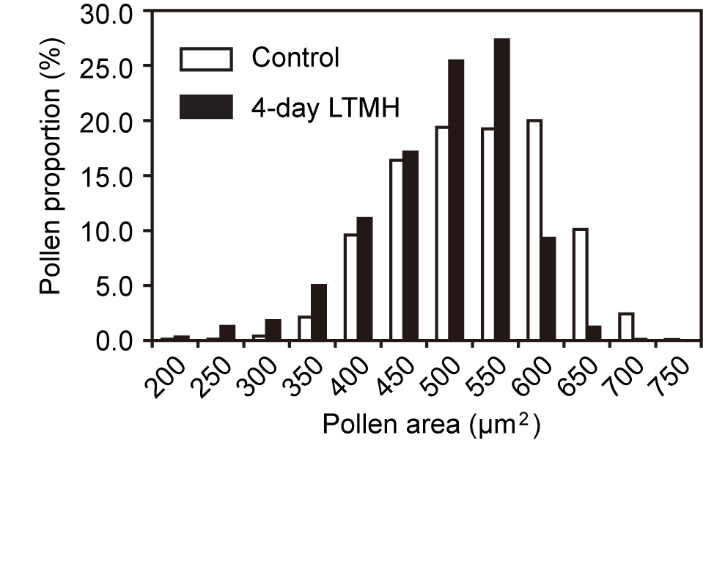


Fig S2. Comparison of the size of anthesis-stage pollen between control and the 4-day long-term mild heat treatment. Pollen area from the 4-day LTMH condition peaked around 500-600 µm^2^ and only 10% of pollen had an area of 600-800 µm^2^, whereas under control temperature, 33% of pollen had an area of 600-800 µm^2^.


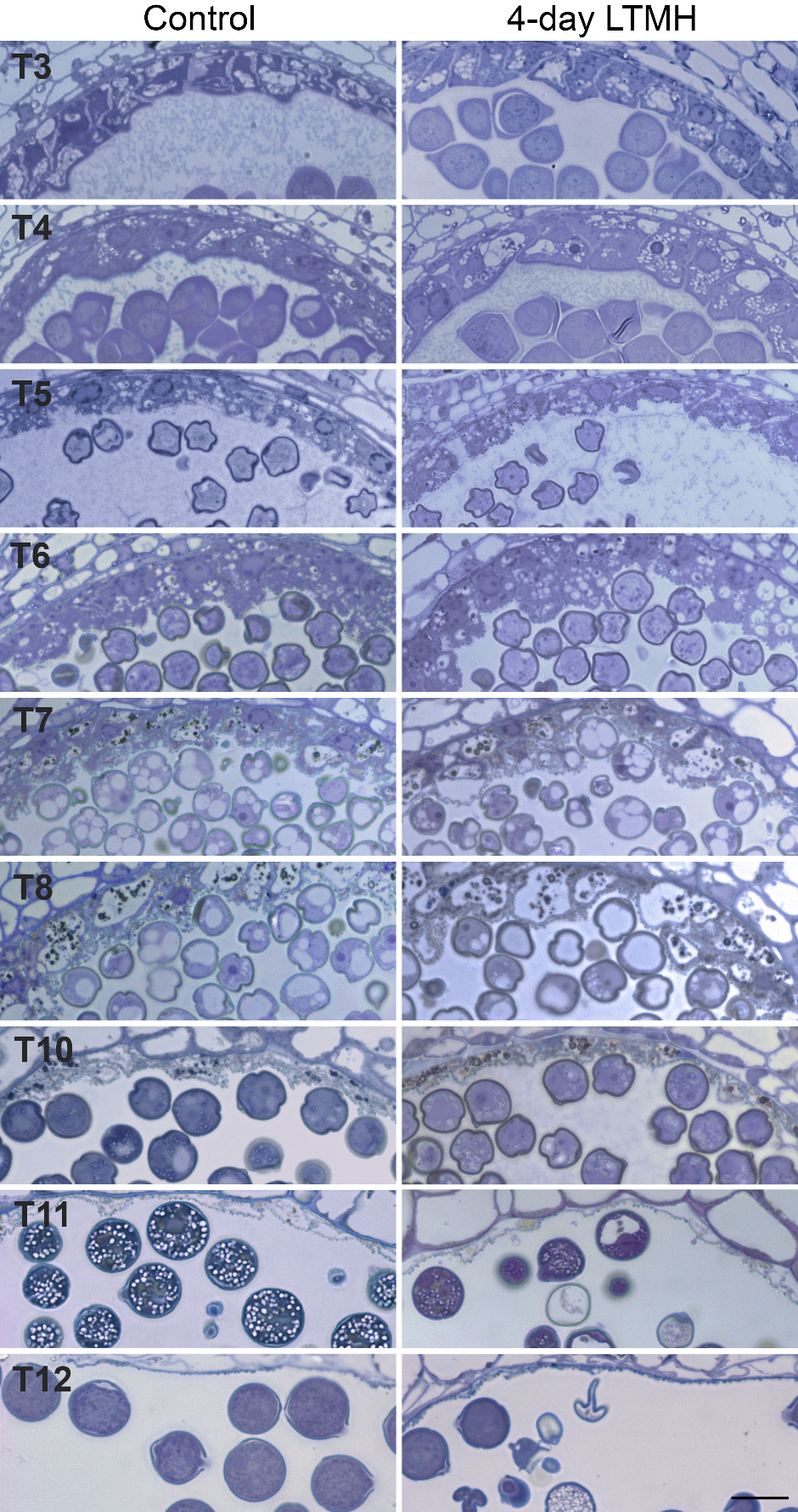


**Fig S3. Cytological comparison of tapetum development between control and 4-day long-term mild heat treatment.** Long-term mild heat (LTMH) or control temperature were applied for 4 days, and flowers that were at the 10-DBA stage on the last day of the treatment (i.e. they were treated from 13 DBA to 10 DBA) were labelled and then sampled on subsequent days for cytological analysis. Examples of tapetum stages T3-T12 (Table S2) are shown, with samples from the two treatments always derived from the same day after treatment and with comparable pollen developmental stage. For quantitative representation see Fig 5.


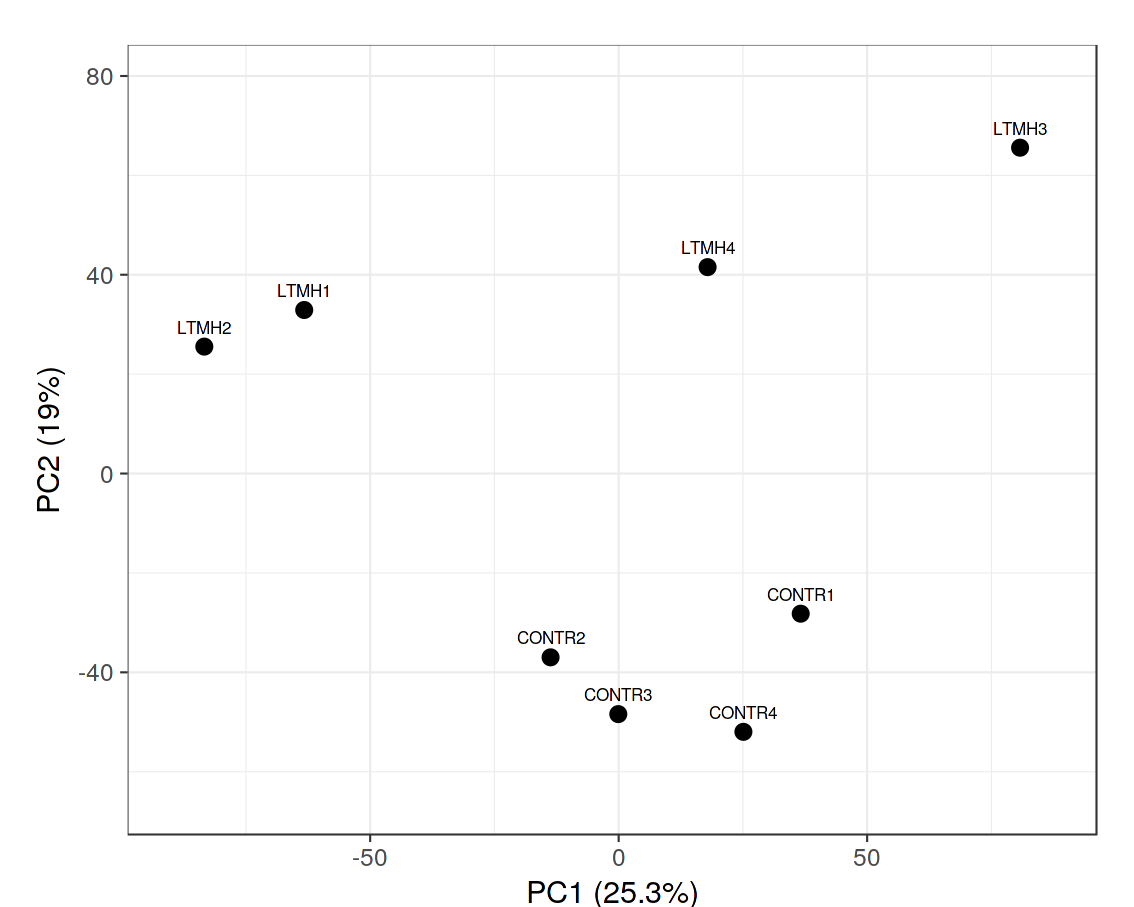


**Fig S4. Principle component analysis of individual transcript profiling samples.** Samples derived from control (CONTR) or 4-day long-term mild heat (LTMH) treatments.


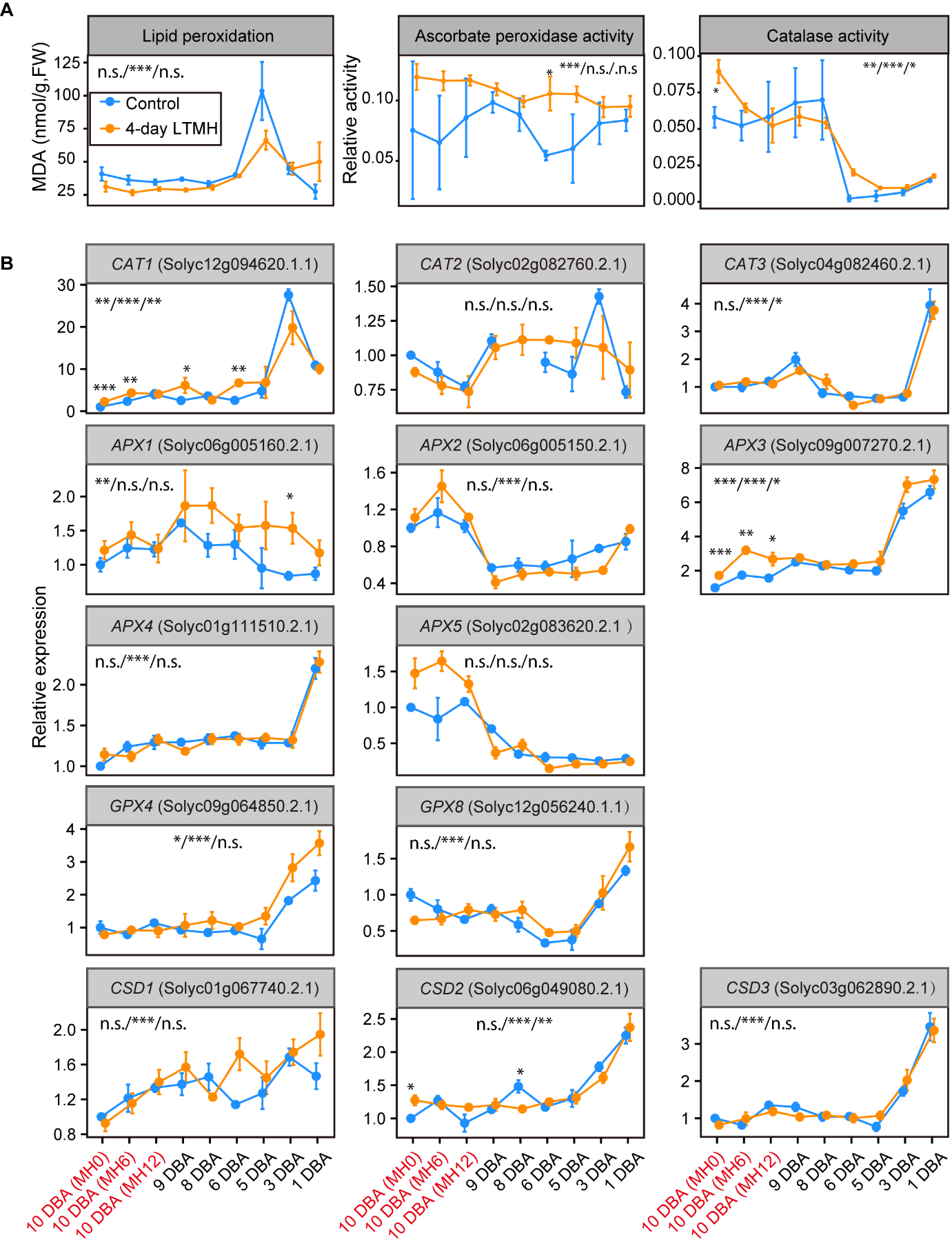


**Fig S5. Analysis of ROS scavenger activity and gene expression in anthers upon 4-day long-term mild heat treatment.** Analysis was done during the fourth day of control (CT) or long-term mild heat (LTMH) treatment (10 DBA; 3 time points) and on subsequent days. A) MDA level, ascorbate peroxidase activity and catalase activity in anthers. B) Transcript levels of tomato catalase, ascorbate peroxidase, superoxide dismutase and glutathione peroxidase genes in anthers. Values are the mean ±SE (n=3 plants for A and 4 plants for B, in each case with pools of 1-4 anther cones per plant). Significance of main effects of temperature treatment, time point and their interaction, respectively, are indicated in each pane. *, significant effect (two-way ANOVA) or significantly different between treatments (one-way ANOVA with LSD), P≤0.05; **, P≤0.01; ***, P≤0.001. n.s., not significant.
